# Modeling early recovery of physical function following hip and knee arthroplasty

**DOI:** 10.1186/1471-2474-7-100

**Published:** 2006-12-11

**Authors:** Deborah M Kennedy, Paul W Stratford, Steven E Hanna, Jean Wessel, Jeffrey D Gollish

**Affiliations:** 1 School of Rehabilitation Science, McMaster University, Hamilton, ON, Canada; 2 Centre for Studies of Physical Function, Sunnybrook Holland Orthopaedic and Arthritic Centre. Department of Physical Therapy, University of Toronto, Toronto, ON, Canada; 3 Department of Clinical Epidemiology and Biostatistics, McMaster University, Hamilton, ON, Canada; 4 Division of Orthopaedic Surgery, Sunnybrook Health Sciences Centre. Department of Surgery, Faculty of Medicine, University of Toronto, Toronto, ON, Canada

## Abstract

**Background:**

Information on early recovery after arthroplasty is needed to help benchmark progress and make appropriate decisions concerning patient rehabilitation needs. The purpose of this study was to model early recovery of physical function in patients undergoing total hip (THA) and knee (TKA) arthroplasty, using physical performance and self-report measures.

**Methods:**

A sample of convenience of 152 subjects completed testing, of which 69 (mean age: 66.77 ± 8.23 years) underwent THA and 83 (mean age: 60.25 ± 11.19 years) TKA. Postoperatively, patients were treated using standardized care pathways and rehabilitation protocols. Using a repeated measures design, patients were assessed at multiple time points over the first four postoperative months. Outcome measures included the Lower Extremity Function Scale (LEFS), the physical function subscale of the Western Ontario and McMaster Universities Osteoarthritis Index (WOMAC PF), the 6 minute walk test (6 MWT), timed up and go test (TUG) and a timed stair test (ST). Average recovery curves for each of the measures were characterized using hierarchical linear modeling. Predictors of recovery were sequentially modeled after validation of the basic developmental models.

**Results:**

Slopes of recovery were greater in the first 6 to 9 weeks with a second-degree polynomial growth term (weeks squared) providing a reasonable fit for the data over the study interval. Different patterns of recovery were observed between the self-report measures of physical function and the performance measures. In contrast to the models for the WOMAC PF and the LEFS, site of arthroplasty was a significant predictor (p = 0.001) in all of the physical performance measure models with the patients post TKA initially demonstrating higher function. Site of arthroplasty (p = 0.025) also predicted the rate of change for patients post THA and between 9 to 11 weeks after surgery, the THA group surpassed the function of the patients post TKA.

**Conclusion:**

Knowledge about the predicted growth curves will assist clinicians in referencing patient progress, and determining the critical time points for measuring change. The study has contributed further evidence to highlight the benefit of using physical performance measures to learn about the patients' actual level of disability.

## Background

As the demand for total hip (THA) and knee arthroplasty (TKA) continues to increase, there will be an associated increase in the need for rehabilitation services. Most patients receive some form of postoperative rehabilitation services through inpatient rehabilitation, home care or outpatient services [1]. Because of limited resources, health care professionals need information on early recovery in the first few months after surgery to help them benchmark progress and make appropriate decisions concerning patient rehabilitation needs.

There is no consensus on the best outcome measures to monitor recovery following arthroplasty. Many of the studies examining postoperative recovery [2-6] use only self-report condition specific or generic health status measures. In addition, many studies do not specifically measure change at intervals during the early period of greatest change after surgery [7]. A systematic review of the literature reported the Medical Outcomes Study Short Form-36 (SF-36) and the Western Ontario and McMaster Universities Osteoarthritis Index (WOMAC) were the most frequently used instruments. Evaluations typically occurred 6 to 12 months post THA and TKA [8].

Prior research has shown that at best, only a moderate correlation exists between self-report and performance measures [9-12]. Performance measures may detect changes in physical function that self-report measures do not. Using the WOMAC physical function subscale, SF-36 physical function and role-physical subscales, stair ascent cycle duration, gait speed over 10 meters and the six-minute walk test, Parent and Moffet [7] assessed patients after TKA, preoperatively and at 2 and 4 months postoperatively. At 2 months, the WOMAC physical function subscale and SF-36 physical function domain demonstrated significant improvement compared to patients' preoperative values whereas the locomotor tests were comparable to their preoperative values or significantly less (six-minute walk distance). Previous studies by the authors have similarly noted that the physical function subscale of the WOMAC has failed to detect changes in physical function that were evident with performance assessment [13,14]. The results of these studies suggest that self-report measures comment more on a patient's experience of moving around whereas performance specific measures may better represent the ability to move around.

Although the primary goals of arthroplasty are to decrease pain and improve function, there has been an increasing emphasis on the recovery of function particularly with the technological improvements of the implants [15,16]. Because of the paucity of longitudinal studies evaluating the early time course of physical functional change at frequent intervals, the purpose of the current study was to characterize early recovery using a combination of self-report and physical performance measures in patients following THA and TKA. The analytic approach of hierarchical linear modeling (HLM) was chosen as it provides a flexible technique for dealing with longitudinal data [17,18]. Using HLM, the specific purposes of the study were to model recovery curves for the Lower Extremity Function Scale (LEFS), the physical function subscale of the WOMAC (WOMAC PF), the 6-minute walk test (6 MWT), the timed up and go test (TUG) and a timed stair test (ST) and to compare differences in their patterns of recovery. The models provide the scores and rate of change of the scores for the outcome measure of interest. Clinicians, researchers, and perhaps even health policy makers, can apply this information to make decisions concerning the optimal assessment intervals and to predict recovery post arthroplasty.

## Methods

### Design

The current investigation was part of a prospective, observational study that applied a repeated measures design to examine recovery during the first four months following arthroplasty surgery. Ethics approval for the study was received from the institution's research ethics review board and all participating patients provided written informed consent. All patients underwent a baseline preoperative visit one to two weeks prior to surgery and received standardized inpatient treatment, following either a primary total hip or knee care pathway.

In terms of the knee prostheses, all were cemented and posterior-stabilized. Patients undergoing TKA were permitted to be full weight bearing and participated in a progressive program of range of motion, strengthening exercises, proprioceptive exercises and functional training. Uncemented or hybrid configurations were used with respect to the hip prostheses. Patients following THA were educated about postoperative movement restrictions such as the avoidance of hip flexion beyond 90 degrees, hip adduction beyond neutral and excessive hip rotation for the first 6–8 weeks. Hip range of motion, and strengthening exercises were initiated along with functional training. At the time of this study, 66% of the patients post THA had weight bearing restrictions. Most of the patients (83%) were transferred from the acute care floor to the onsite short-term rehabilitation unit to continue to progress the aforementioned programs for a maximum length of stay of 7 days.

To provide a more accurate model of change over time, follow-up assessments were not conducted at exactly the same time points. If all measurements are performed at the same points in time, change can only be detected at these assessment points. The first assessment in most cases occurred at discharge from hospital and subsequent visits were often scheduled in conjunction with surgeon follow-up appointments. Typically these occurred around 6 weeks and 3 months, however, additional assessments were conducted before and after these time points.

### Subjects

Subjects were recruited from a specialized, orthopaedic tertiary care facility with high volumes of lower extremity arthroplasty. Inclusion criteria were as follows: a diagnosis of osteoarthritis (OA), scheduled for primary total joint arthroplasty, sufficient language skills to communicate in written and spoken English, and absence of neurological, cardiac, psychiatric disorders or other medical conditions that would significantly compromise physical function. Patients who underwent revision, bilateral or staged arthroplasties were excluded.

One hundred eighty-eight patients provided informed consent between October 2001 and March 2003. Thirty-six patients had only baseline assessments and were lost to follow-up for the following reasons: canceled surgery, loss of follow-up due to the outbreak of Severe Acute Respiratory Syndrome (SARS) in Toronto from April to June 2003, additional operative procedures, postoperative medical reasons or patient choice to discontinue. In terms of SARS, on March 26^th^, 2003 SARS was declared a provincial (Ontario) emergency. At the height of the outbreak, thousands of people, mostly in the Toronto area were quarantined for 10 day periods and there were significant restrictions on patient related activities in hospitals. Table 1 provides a summary of the demographic and preoperative baseline characteristics of the 152 (81%) patients who participated.

### Measures

To assist clinicians in benchmarking recovery, commonly used performance measures, the 6 MWT [7,19-22], TUG [23-26] and the ST [11,26,27]were chosen. In a different, preoperative arm of this longitudinal study, a sample of patients while they were on the waiting list was used to establish the reliability of these measures and their ability to detect change following THA and TKA [28]. As some of these patients were followed postoperatively, there may have been some overlap of the samples.

Two self-report measures of functional status, the physical function subscale of the WOMAC [29-32] and the LEFS [33,34]were additionally selected. Historically, the WOMAC has been considered one of the leading health status measures in the arthroplasty population. The LEFS, a region specific measure, was conceived to assess the lower extremity functional status of patients and the reliability estimates have been high when investigated in the THA and TKA population [34]. Using a construct validation approach, the measure has demonstrated cross-sectional and longitudinal validity equal to or better than the WOMAC PF subscale [14].

To complete the 6 MWT, patients were instructed to cover as much distance as possible during the 6 minute time frame with opportunity to stop and rest if required. A pre-measured 46 meter uncarpeted, rectangular circuit was marked off in meters and the distance each subject covered was measured to the nearest meter. Standardized encouragement was given at 60 second intervals as previous work has demonstrated that encouragement improves performance [35].

The TUG [36] was performed by having study participants rise from a standard armchair, walk at a safe and comfortable pace to a tape mark 3-meters away and then return to a sitting position in the chair. A standardized script was read to each patient and the test began on the word "go".

The timed stair test required the participants to ascend and descend one flight of nine stairs in their usual manner, at a safe and comfortable pace. For all of the performance measures, time was measured to the nearest one hundredth of a second using a stopwatch and subjects were instructed to use their regular walking aids as needed.

Because the focus of the current study was on physical function, the results of the WOMAC physical function subscale are presented, however patients completed the pain, stiffness and physical function subscale of the WOMAC version LK3.1. The physical function subscale has 17 items, which are scored on a scale of 0 to 4 (total score 68), with higher scores representing greater difficulty performing the activity.

The LEFS is composed of 20 items each scored on a 5 point adjectival scale with '0' extreme difficulty or unable to perform the activity and '4' no difficulty.^12 ^The items are summed to produce a total LEFS score, which can vary from 0 to 80. The LEFS has the opposite orientation to the WOMAC with higher total LEFS' scores signifying better lower extremity functional status.

### Analysis

All analyses were conducted using SPSS version 11.5 statistical software. Descriptive statistics were calculated for each of the measures according to site of arthroplasty. The baseline performance and other characteristics of participants who withdrew were compared to those participants who completed testing using independent t-tests. T-tests were also used to compare baseline performance measure scores between participants who could or could not complete testing at discharge from hospital.

Hierarchical linear modeling also known as linear mixed-effects modeling or multilevel modeling, was used to characterize the average pattern of recovery for each of the measures [17,37,38]. The terms "hierarchical" and "multilevel" indicate that variation in the outcome, organized at two or more levels, is being modeled. In the case of repeated measures studies such as this one, it is concerned with the clustering of repeated measurements within the same subject. In addition to estimating average change over time, HLM produces estimates of the degree of individual differences in the pattern of change. A mixed effects approach is used in which some parameters have both fixed and random effects. The fixed effect parameters describe the average change in the population of interest and the random effects describe the individual differences.

To aid in the interpretation of the intercept, the data were centered at one week after surgery as this corresponded to the time when many patients had their first assessment and represented their lowest point of function. Before modeling growth for the TUG measure, a natural logarithmic transformation of the TUG scores was required to correct the positive skew of the data. Several patients had very slow TUG times; however, inspection of the data revealed no basis for excluding them from the analysis.

The basic model of change in physical function over time includes parameters that estimate the patients' self-reported or actual measured function at one week post surgery (intercept) and the patients' rate of change (slope) in function for every week thereafter. The model also includes the square of time as a predictor to estimate the change in the recovery rate for each week after one week. In HLM analyses, individual differences in the recovery pattern are estimated as variances in the parameters of change. The square root of these variances provides an estimate of individual differences in standard deviations.

After validation of the basic recovery model, clinical predictors of recovery were explored. The following were investigated as predictors: gender, site of arthroplasty (hip or knee), additional outpatient rehabilitation after hospital discharge (yes, no), age, body mass index, number of comorbidities and baseline preoperative function. Additional outpatient treatment was investigated because of its potential to facilitate recovery. The variable, number of comorbidities was recoded into a binary variable, < 3 comorbidities or ≥ 3 comorbidities. Age, body mass index and baseline preoperative function were modeled as continuous variables. In each case, the baseline preoperative scores of the outcome measure being modeled were used.

Predictors were explored one at a time, sequentially. Due to the evidence in the literature regarding the influence of gender, baseline preoperative function and site of arthroplasty, these predictors were investigated early in the modeling [39]. When a predictor was added, initially its effect on the average overall score (i.e. the intercept) was evaluated and this was followed by an examination of its interactions with time to test the predictor's effects on the rate of recovery. To clarify the sequential model building, if a predictor was found to have a significant fixed and/or interaction effect, these term(s) would be retained in the model at which point the next predictor would be evaluated. At each step, terms introduced earlier in the modeling would be retained if they were significant. The final multivariate model was determined once all of the predictors had been explored. Plots of the residuals of this model were then assessed to ensure that they were normally and independently distributed with a mean of '0' and constant variance [38]. In addition, plots of the predicted scores for each of the measures were plotted against the actual scores with a linear distribution indicating good fit.

## Results

Independent t-tests comparing the participants to those lost to follow-up revealed that the latter group was significantly older. Although there were no significant differences between the self-reported physical function between these groups, the non-participants exhibited worse function in terms of the performance measures; with respect to the ST and possibly the 6 MWT, p < 0.057 (Table 2).

Of the 152 subjects who participated, 69 underwent THA and 83 TKA. Seventy-five of the subjects were women with 44 of these undergoing TKA and 31, THA. In terms of postoperative complications, 2 patients required blood transfusions, 3 developed urinary tract infections, one developed ileus and one patient within the TKA group had a documented deep vein thrombosis. The patients undergoing TKA were significantly older and had a greater body mass index (Table 1). No significant differences were noted in their baseline function with respect to the five measures.

All participants completed baseline preoperative assessments and had a minimum of at least one assessment during the 4 month postoperative period with 51%, 26% and 6% assessed 2, 3 and 4 times respectively. At discharge from hospital, 33% of the subjects were unable to complete the 6 MWT and 26.0% the ST, compared to only 3% who could not complete the TUG. An independent t-test revealed no significant difference (p = 0.181) between the baseline scores of the individuals who completed the 6 MWT versus those who did not, however, this was not the case with the ST. Individuals who completed the ST at discharge were significantly faster (p = 0.048) at baseline than those not completing it at the discharge assessment point.

**Figure 1:**
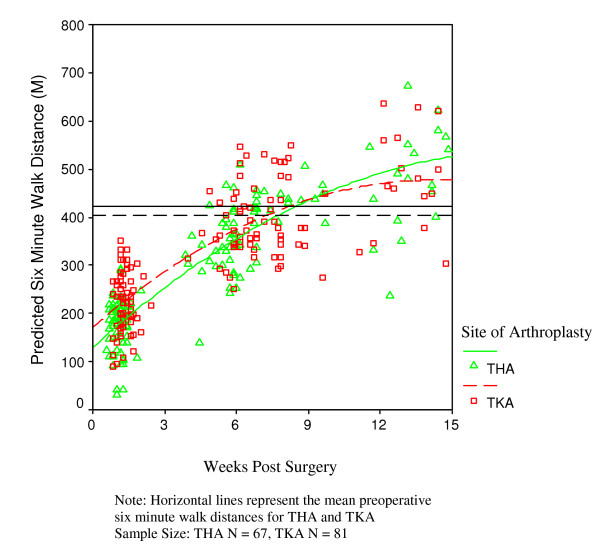
Predicted Recovery Curves for the Six Minute Walk Test According to Site of Arthroplasty.

**Figure 2:**
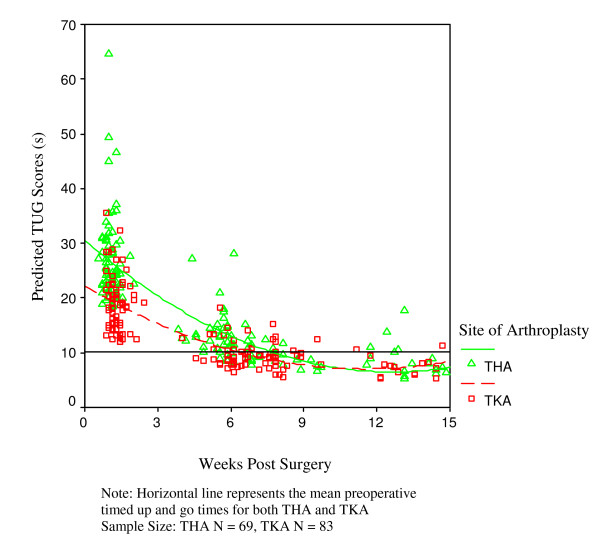
Predicted Recovery Curves for the Timed Up and Go According to Site of Arthroplasty.

**Figure 3:**
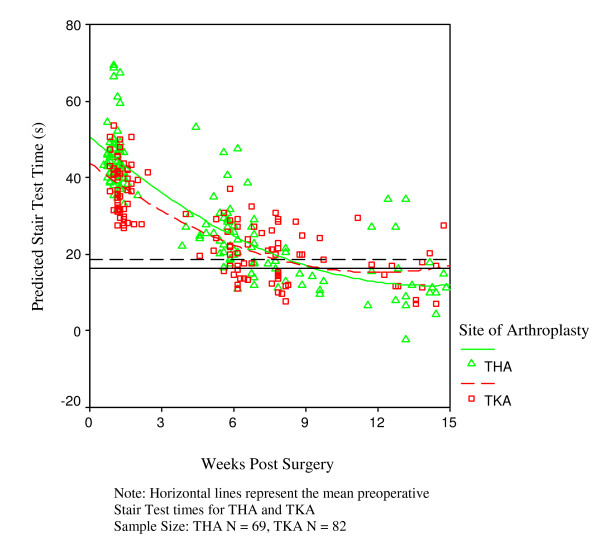
Predicted Recovery Curves for the Stair Test According to Site of Arthroplasty.

**Figure 4:**
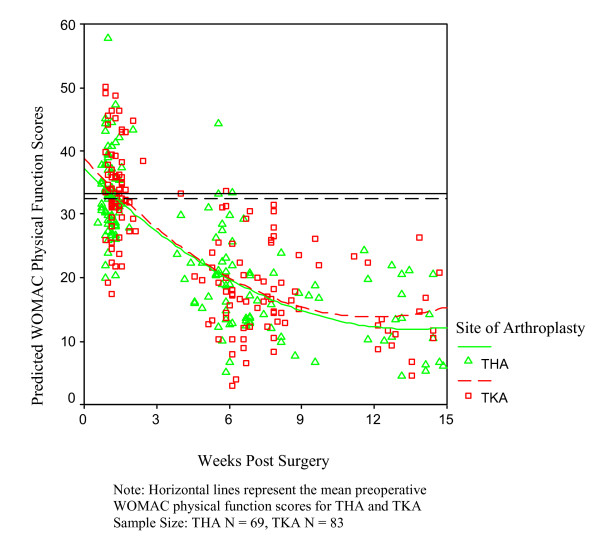
Predicted Recovery Curves for the WOMAC Physical Function Subscale According to Site of Arthroplasty.

**Figure 5:**
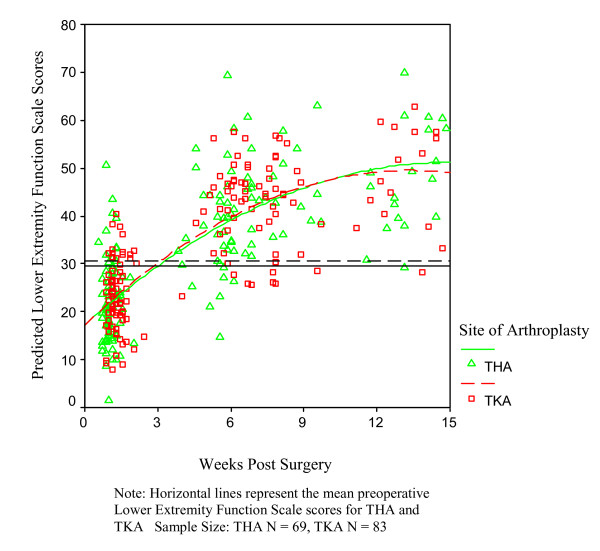
Predicted Recovery Curves for the Lower Extremity Function Scale According to Site of Arthroplasty.

In each of the Figures (1, 2, 3, 4, 5), the graphs were constructed from the measure scores predicted by each of the final developed models. The preoperative mean of each of the measures was plotted as a horizontal line to assist in referencing recovery. Lower scores represent improvement for the WOMAC PF scores, the TUG and the ST with the reverse orientation for the 6 MWT and LEFS. As can be seen in each of the figures, the slopes of recovery were greater with significant change happening in the first 6 to 9 weeks for each of the measures. Different patterns of recovery, however, were observed between the self-report measures of physical function and the performance measures. Patients achieved their mean preoperative WOMAC PF scores by 1–2 weeks and their mean LEFS preoperative scores around 3 weeks. In contrast, patients generally met their mean preoperative scores for the performance measures later between 6 weeks and just over 9 weeks.

The performance measure graphs all demonstrated differences in the recovery pattern for THA and TKA. In each of these models, site of arthroplasty was a significant predictor of the one week scores with the patients post TKA initially demonstrating higher function. However, a significant interaction (in Figures 1, 2, 3, demonstrated at the point at which the lines cross) was observed between the rate of change, weeks after surgery and site of arthroplasty with the patients post THA surpassing the function of the patients post TKA between 9 to 11 weeks. Figure 2 also demonstrates a ceiling effect (lower scores are better) for the TUG measure around nine to ten weeks. Unlike the performance measures, similar patterns of recovery are observed for the participants post THA and TKA with respect to the recovery figures for the WOMAC physical function scores (Figure 4) and the LEFS (Figure 5).

Table 3 provides a summary of the significant fixed effects and random effects for the models with specific model parameter estimates for the 6 MWT and WOMAC PF displayed as samples in Tables 4 and 5. A more detailed examination of the findings with respect to the predictor variables is published elsewhere [39]. In most models, the only significant random effect was the intercept indicating that individuals varied in their starting points one week after surgery; however, their slopes and quadratic component of time were similar. The LEFS model was the exception with both the intercept and slope significant. To understand how the standard deviations of the intercepts give an indication of between-patient differences in functioning, consider the results for the 6 MWT reported in Table 4. The mean distance at 1 week is 142.43, but the standard deviation of the intercepts is estimated to be 53.70. According to the standard model assumptions, about two thirds of patients are expected to have scores +/- 1 SD from the mean. Therefore, the variations in 6 MWT are such that about two thirds of patients' 1-week scores are expected to be in the range of 88.7 to 196.1 meters. Since these estimates are from the final multivariate model, this suggests that large variations in short-term post operative function remain, even after accounting for the available clinical predictors.

To further clarify the interpretation of the interaction terms as estimates of the effects of the predictors on the amount of change over time, one can refer to the estimates in Table 4. The estimate for the interaction of weeks after surgery with baseline 6 MWT score is .036 indicating that each increase of 1 meter in baseline score, predicts more positive recovery of .036 meters per week. Therefore, if an individual had a baseline score which exceeded another by 100 meters, this would translate into 36 more meters at 10 weeks postoperatively. In the five models, slowing in the recovery trajectory occurred over time and in all models, a second-degree polynomial growth term (quadratic-weeks squared) provided a reasonable fit for the data over the study interval.

## Discussion

This study has helped to address the paucity of longitudinal studies examining recovery, using physical performance and self-report measures, during the period of greatest change after THA and TKA [7,24,40]. Trajectories of recovery out to 15 weeks postoperatively have been established with a straightforward model providing a reasonable fit for the data over this interval. However, based on the characteristics of this particular kind of model (involving the quadratic component of time), the results should not be extrapolated beyond the time points used to generate the model. This includes not interpreting the values at zero weeks because initial testing began approximately one week after surgery.

Using hierarchical linear modeling, this study has contributed evidence that different and important information can be learned from administering physical performance measures to assess physical function. In each of the performance measure models, site of arthroplasty was a predictor of one week scores indicating that patients post TKA began with higher function. A significant interaction with site of arthroplasty and weeks after surgery was also apparent in each of the 6 MWT, TUG and ST models. Although patients undergoing THA started postoperatively with worse function, their rate of recovery was faster than their knee counterparts with respect to each of the performance measures. In contrast, the growth curves for the LEFS and physical function subscale of the WOMAC were not significantly different following THA and TKA. In the graphs (Figures 1, 2, 3), the patients post THA catch and surpass those following TKA around 9 to 10 weeks. This is likely a reflection of the cessation of the early postoperative hip restrictions beginning at 6 weeks. Another contributing factor would have been the postoperative weight bearing status of the patients following THA with a significant proportion of the patients progressing from restricted to full weight bearing at the 6 week mark.

A striking difference between the self-report measures of physical function and performance measure graphs concerns the point at which preoperative scores are predicted to be met. The predicted scores for the WOMAC physical function subscale met the preoperative scores at one to two weeks (Figure 4) and the preoperative LEFS scores were met around 3 weeks (Figure 5). In contrast the preoperative 6 MWT distances were met between 7 – 8 weeks, the TUG between 6 – 8 weeks and the ST around 8 to 10 weeks. It would appear that the WOMAC physical function subscale is not reflecting the early deterioration that occurs in physical function. Consistent with findings from an earlier study [14], the LEFS did reflect some of the early deterioration. Walker et al [41]reported a similar finding when comparing measured ambulatory activity to self-reported data in patients post TKR. Their results indicated that self-reported mobility improved before the subjects were actually doing more. Other authors have noted similar findings [7,24,26]. It has been suggested that physical performance measures of functioning may confer advantages over self-report measures in the evaluation of change [42,43].

The recovery curves for each of the measures will serve as helpful guides for clinicians faced with the decision of choosing the most informative measures. The predicted growth curves for the TUG confirm its usefulness only in the early recovery period after arthroplasty. Around 9 to 10 weeks there is a ceiling effect. At this time, both the THR and TKR groups having passed the benchmark of 10 seconds which has been documented to be the level at which patients are functionally independent [36]. Reference TUG scores for community-dwelling elderly people with independent functioning further confirm this finding [44]. The mean TUG score has been reported to be 8 seconds for the age group 60 to 69 and 9 seconds for 70 to 79 year olds [44]. Mizner et al [24] similarly found no change in TUG scores between 3 and 6 months in a group of patients who underwent TKA followed by rehabilitation. As noted in the results, the TUG is a measure that most patients can complete at discharge from hospital and can be of benefit to clinicians administering treatment in the early postoperative period. In contrast, the 6 MWT and ST would not be the best measures for the early postoperative period as in this study between 26–33% of patients could not complete them at discharge. With the exception of the TUG measure, further study will be required to examine the recovery curves of the 6 MWT and ST to determine how much further improvement in function is obtained and to determine when patients have reached the stage of most benefit from surgery.

The recovery curves also facilitate determination of the critical time points for measuring change. Stratford has demonstrated that the ideal assessment interval for any evaluative measure occurs when 50% of patients achieve a change equal to or greater than the minimal detectable change (MDC) [45]. By combining the information provided by the growth curve and knowledge of the MDC, the time for reassessment can be planned to minimize the measurement error associated with too frequent or infrequent follow-up. MDC values for each of the measures profiled in this study have been published [28,34,46].

One limitation in the current study was missing data across some of the time points. As reported earlier, only 83% of the patients had two or more visits. It was planned that patients would have a minimum of 2 visits but preferably 3 in the first 4 months, although the scheduled times were not to be standardized. The fact that more than 50% of the subjects had only two measurements could have impacted the modeling of the quadratic time component as three time points are required. An important advantage of hierarchical linear modeling, however, is that the number and timing of observations need not be the same across all subjects [17,47]. In the case of the patients who had limited data, the mixed effects models would stabilize their estimates by anchoring them to the group average. However, bias will still result if the cause of the missingness is related to the outcome that would have been observed. For example, this could have been a problem in the case of the 26% of patients who were unable to complete the ST at their discharge from the hospital. As noted in the results, this group of patients was slower than their counterparts preoperatively and they may have deferred testing due to their postoperative acuity. As a result, had they been tested they might have contributed slower ST times and the absence of their scores could have led to overestimation of the growth curves at the one-two week mark. This may have also been the case with the 6 MWT predicted scores corresponding to the discharge assessment.

Another consequence of the limited data points was its potential impact on the random effects of the recovery curves. Having fewer data points restricts the complexity of the random effects possible. With the exception of the LEFS model, the only significant random effect was the intercept, indicating that individuals varied in their starting point one week after surgery. In the case of the LEFS, there was a random effect for the growth parameter weeks after surgery, meaning that individuals varied in their rate of recovery.

A further limitation was the sample. Individuals with the highest disability would not have participated because of the nature of the performance measurements. This could have impacted the estimation of the recovery curves.

## Conclusion

Knowledge about the predicted growth curves for the 6 MWT, TUG, ST, WOMAC physical function subscale and LEFS will assist clinicians in monitoring progress at the appropriate time periods and will ultimately facilitate enhanced treatment decision making along the continuum of care. Depending on the time period of administration, the recovery curves also provide information about the choice of measure with the TUG useful only in the early postoperative period. The study has contributed further evidence to highlight the benefit of using physical performance measures to assess recovery of physical function post arthroplasty as important information is gained about the patient's actual level of disability.

## Competing interests

The author(s) declare that they have no competing interests.

## Authors' contributions

DMK conceived and designed the study, assisted with the data collection, performed the statistical analysis and prepared the manuscript.

PWS assisted with the design, statistical analysis/interpretation and manuscript preparation.

SEH assisted with the design, statistical analysis/interpretation and manuscript preparation.

JW consulted in the conception and design of the study and assisted with the manuscript preparation.

JDG assisted with the design and execution of the study and manuscript preparation.

All authors read and approved the final manuscript.

## Pre-publication history

The pre-publication history for this paper can be accessed here:



## Figures and Tables

**Table 1:** Demographic and Preoperative Functional Scores by Site of Replacement

**Patient Characteristics And Baseline, Preoperative Function**	**TKA Mean, (SD), N**	**THA Mean (SD) N**	**T-Test Results p-value**
Age (years)	66.77, (8.23), 83	60.25, (11.19), 69	p < 0.001
BMI kg/m^2^	31.42, (4.69), 83	28.60, (4.56), 68	p < 0.001
TUG score (seconds)	10.15, (3.66), 83	10.14, (3.71), 69	p = 0.987
6 MWT distance (meters)	403.07, (120.10), 81	422.90, (115.65), 67	p = 0.311
Stair Test (seconds)	18.69, (8.37), 82	16.41, (11.42), 69	p = 0.160
WOMAC PF score	32.47, (11.89), 83	33.32, (10.60), 69	p = 0.646
LEFS score	30.51, (12.44), 83	29.45, (12.73), 69	p = 0.607

Abbreviations: TKA = Total Knee Arthroplasty

THA = Total Hip Arthroplasty

N = Number

SD = Standard Deviation

BMI = Body Mass Index

TUG = Timed up and go test

6 MWT = Six Minute Walk Test

PF = Physical Function

LEFS = Lower Extremity Function Scale

**Table 2:** Summary of Patient Characteristics and Baseline Preoperative Function

**Patient Characteristics And Baseline, Preoperative Function**	**Dropouts/Withdrawn Patients Mean, (SD), N**	**Subjects Mean (SD) N**	**T-Test Results p-value**
Age (years)	68.03, (12.30), 36	63.81, (10.19), 152	p = 0.033
BMI kg/m^2^	29.96, (5.40), 36	30.15, (4.82), 151	p = 0.839
TUG score (seconds)	11.23, (4.39), 36	10.14, (3.67), 152	p = 0.128
6 MWT distance (meters)	370.06, (111.20), 35	412.05, (118.12), 148	p = 0.057
Stair Test (seconds)	22.44, (9.34), 35	17.65, (9.91), 151	p < 0.010
WOMAC PF score	30.97, (12.19), 36	32.86, (11.30), 152	p = 0.377
LEFS score	32.61, (13.21), 36	30.03, (12.54), 152	p = 0.272

Abbreviations: N = Number

SD = Standard Deviation

BMI = Body Mass Index

TUG = Timed up and go test

6 MWT = Six Minute Walk Test

PF = Physical Function

LEFS = Lower Extremity Function Scale

**Table 3:** Significant Fixed Effects and Random Effects for Each of the Models

Measure	Average Recovery Model (Fixed Effects)	Individual Variations in Recovery (Random Effects)
Six Minute Walk test (6 MWT)	intercept (one week status), slope (rate of change at 1 week), quadratic term (weeks^2^) **Predictors: **preoperative 6 MWT baseline score, gender; site of arthroplasty **Interactions: **site of arthroplasty with rate of change at 1 week, baseline score with rate of change at 1 week	intercept
Timed Up and Go Test (TUG)	intercept, slope, quadratic term **Predictors**: preoperative TUG baseline score, gender, site of arthroplasty, additional outpatient treatment **Interactions: **site of arthroplasty with rate of change at 1 week	intercept
Timed Stair Test (ST)	intercept, slope, quadratic term **Predictors: **preoperative ST baseline score, gender, site of arthroplasty, additional outpatient treatment **Interaction**s: site of arthroplasty with rate of change at 1 week	intercept
WOMAC Physical Function (PF) Subscale	intercept, slope, quadratic term **Predictors: **preoperative WOMAC PF baseline score	intercept
Lower Extremity Function Scale	intercept, slope, quadratic term **Predictors**: preoperative LEFS baseline score, gender	intercept, slope

**Table 4:** Model Parameter Estimates for the Six Minute Walk Test

	**Parameter**	**Six Minute Walk Test Estimate (95% CI) Level of Significance**
	
**Fixed Effect Estimates**	Intercept (mean distance in meters at 1 week)	142.34 (80.93, 203.76) p < .0005
	Slope (weeks after surgery)	32.05 (24.84, 39.26) p < .0005
	Quadratic (weeks)^2^	-1.70 (-2.03, -1.37) p < .0005
	6 MWT Baseline Score	.126 (.0002, .253) p = .05
	Interaction of weeks after surgery by baseline score	.036 (.023, .048) p < .0005
	Gender	If female, -60.12 (-85.69, -34.55) p < .0005
	Site of arthroplasty (THA or TKA)	If TKA, 48.30 (19.91, 76.68) p = .001
	Interaction of weeks after surgery by site of arthroplasty	If TKA, -3.70 (-7.05, -.353) p = .030
		**Standard Deviation**
	
**Variance Estimates**	Within Patient Residual	56.20
	Intercept	53.70

Abbreviations: CI, Confidence Interval

6 MWT, Six Minute Walk Test

TKA, Total Knee Arthroplasty

THA, Total Hip Arthroplasty

**Table 5:** Model Parameter Estimates for the WOMAC Physical Function Subscale

	**Parameter**	**WOMAC PF Estimate (95% CI) Level of Significance**
	
**Fixed Effect Estimates**	Intercept (mean score at 1 week)	23.92 (19.21, 28.63) p < .0005
	Slope (weeks after surgery)	-3.62 (-4.17, -3.06) p < .0005
	Quadratic (weeks)^2^	.140 (.100, .181) p < .0005
	WOMAC Physical Function Baseline Score	.319 (.185, .452) p < .0005
		**Standard Deviation**
	
**Variance Estimates**	Within Patient Residual	7.68
	Intercept	7.48

Abbreviations: CI, Confidence Interval

PF, Physical Function

TKA, Total Knee Arthroplasty

THA, Total Hip Arthroplasty
